# Waning immunity to SARS-CoV-2 following vaccination or infection

**DOI:** 10.3389/fmed.2022.972083

**Published:** 2022-10-13

**Authors:** Carlos Hernandez-Suarez, Efrèn Murillo-Zamora

**Affiliations:** ^1^Instituto de Ciencias Tecnología e Innovación, Universidad Francisco Gavidia, San Salvador, El Salvador; ^2^Unidad de Medicina Familiar No. 19, Departamento de Epidemiología, Instituto Mexicano del Seguro Social, Colima, Mexico

**Keywords:** SARS-CoV-2, immunity, vaccines, waning, survival analysis, vaccine efficacy

## Abstract

We use survival analysis to analyze the decay in the protection induced by eight SARS-CoV-2 vaccines using data from 33,418 fully anonymized patients from the IMSS public health system in Mexico, including only previously vaccinated, confirmed SARS-CoV-2 positive with a PCR test. We analyze the waning effect in those with complete vs. incomplete dose fitting a Weibull distribution. We compare these results with an estimate of the waning effect due to active infection. In two-dose vaccines, we found that the average protection time of a complete dose increases 2.6 times compared to that of an incomplete dose. All analyzed vaccines provided a protection that lasted longer than the protection due to active infection, except in those patients that did not fulfilled the complete dose. The average protection of a full dose is 2.2 times larger than that provided by active infection. The average protection of active infection is about the same as the average protection of an incomplete dose. All evaluated vaccines had lost most of their protective effect between 8 and 11 months of application of first shot. Our results highly correlate with NT_50_ and other estimates of vaccine efficacy. We found that on average, vaccination increases Age_50_, the age at which there is a 50% probability of severe disease if infected, in 15 years. We also found that Age_50_ increases with mean protection time.

## 1. Introduction

Several reports suggest that the protective effect of vaccines against SARS-CoV-2 virus wanes with time. Characterizing how immunity wanes is relevant for policy making, especially regarding vaccination strategies (1). These kinds of studies may be useful to design the best interval between doses or booster shots.

Studies that analyze how the vaccine protection decays over time can be classified into two types: those that measure surrogates of humoral response and those that measure vaccine efficacy (VE) at several successive points in time. Among the first category is (2) who observed a decrease in anti-spike antibody titer of 84.3% between months 1 and 6 for Pfizer vaccine, whereas (3) analyzed the cycle threshold (Ct) values of RdRp gene, that initially increased by 2.7 relative to unvaccinated in the first month after the booster dose, but then decayed to 1.3 in the second month and found to be small in the third to fourth months. Healthcare workers—considered to be at higher risk—who received the two doses of the Pfizer vaccine developed protective antibodies that were maintained at detectable levels at least for 250 days after the second dose of the vaccine (4). Other studies that report reduction in humoral response for several vaccines include (5–12).

The estimates of VE are in general *relative risk* measures, some function of attack ratios (13) measured usually through a cohort-study or a test-negative design (14) and thus provide a comparison between relative risk of vaccinated and unvaccinated groups. The estimates of VE are in general relative risk measures, some function of attack ratios (13) measured usually through a cohort-study or a test-negative design (14) and thus provide a comparison between relative risk of vaccinated and unvaccinated groups.

In the simplest VE model, called all/nothing, it is assumed that a fraction θ of vaccinated individuals is unprotected, and thus VE = 1 − θ. If in a population of size *n*_0_ + *n*_1_ individuals *n*_1_ of them are vaccinated with an all/nothing vaccine with efficacy VE = 1 − θ, then, if a fraction *f* of the population is infected, the expected number of infections among the unvaccinated is *n*_0_*f* and that among the vaccinated is *n*_1_*fθ*. The attack ratio among the first group is *f* and that among the second, *fθ*, which is why the ratio of these two attack ratios can be used to estimate θ and from here, the VE. Other models of vaccine action (leaky, leaky/nothing, etc.) can be also constructed intuitively using urn models (15, 16).

Tartof et al. (17) argues that a reduction in vaccine effectiveness against SARS-CoV-2 infections over time is more likely to be due to waning immunity rather than the delta variant escaping vaccine protection. Several studies suggest a significant waning of the VE from 90 days after the second dose (1, 18–21).

Here we attempt to characterize how the vaccine effect wanes, using the observed time from vaccination (first dose) to the time the patient with a confirmed (PCR) SARS-CoV-2 infection, exhibited the first symptoms. In order to reduce the effect of the appearance in January 2022 of the Omicron variant B.1.1.529 on this study, we considered individuals vaccinated before 31/Ago/2021 only. Using the date of first dose as a baseline will allow us to characterize the waning effect in those individuals that received a single dose only, for those vaccines with two recommended doses. There is a main problem in our method of study, which is related to the differential exposure to infection at which individual are subjected, within and between vaccines. This differential exposure is not the random exposure that is normal to individuals in a population, but to the differential deployment of vaccines. Exposure is clearly related to the number of infected individuals in the population, which in turn is reflected in the appearance of “waves.” If a vaccine has a large fraction of individuals with a reduced remaining efficacy at a time when the exposure increases, this will affect the estimate of how much the vaccine effect has waned. This will be discussed later in detail.

In addition, we will use the information provided by repeated infections of non-vaccinated individuals to characterize the waning effect of the immunity to active infection. We use only individuals with two infections and use the elapsed time between two consecutive infections. These results will be compared against the pattern of antibody decay reported in Varona et al. (12).

We characterize how vaccine efficacy wanes with time, by fitting a survival model to the waning effect, using the same model for all vaccines. The advantage of using survival analysis is that the decay of the protective effect is not a relative measure, as it occurs with VE, but instead is a measure of how the protective effect decays independently of the response of the individual when the protection fails. This will allow to propose an index to measure the protective effect of the vaccine. By considering active infection some sort of immunization, we can also calculate our index for those non-vaccinated infected and compare it against vaccination. We will also analyze how our index correlates with the mean neutralization level of antibodies against SARS-CoV-2 (NT_50_) and with some reported measures of VE found in Khoury et al. (7) and Padmanabhan et al. (11). We complete our analysis with a study on the protective effect of the vaccines on disease severity.

## 2. Vaccines included in the study

Table 1 shows the vaccines included in this study, whereas the amount of data available in main database for each vaccine in the first 14 months is shown in Table 2.

**Table 1:** Vaccine name, manufacturer an abbreviation used in this study.

**Vaccine**	**Manufacturer**	**Abbreviation**
ChAdOx1	Oxford/AstraZeneca	AZ
Ad5-nCoV Convidecia	CanSino	CA
mRNA-1273	Moderna Biotech	MO
BBIBP-CorV	Sinopharm	SP
CoronaVac	Sinovac	SV
Sputnik V/Gam-COVID-Vac	Gamaleya	GA
Ad26.COV2. S	Janssen	JA
BNT162b2	Pfizer/BioNTech	PF

**Table 2:** Number of vaccinated individuals exceeding *n* months from their vaccination with a first dose up to 28/Mar/2022.

**Months**	**AZ**	**CA**	**MO**	**SP**	**SV**	**GA**	**JA**	**PF**
3	499,528	64,681	24,111	6,925	129,441	89,351	60,861	354,110
4	495,515	64,139	23,927	6,912	129,105	88,748	60,723	349,870
5	485,542	62,466	23,337	6,848	127,840	86,386	60,465	344,890
6	467,213	58,711	21,441	6,644	125,222	72,846	60,104	338,373
7	432,815	56,224	11,496	6,308	119,469	62,808	59,279	321,107
8	344,027	50,940	5,657	4,817	91,977	56,368	57,670	292,447
9	191,611	45,854	3,696	2,738	47,583	31,549	51,998	246,435
10	96,637	41,605	2,552	1,839	30,490	23,232	7,478	202,420
11	47,542	22,493	1,497	1,137	17,783	11,942	1,160	147,750
12	25,326	6,704	802	812	11,923	6,466	602	116,041
13	12,125	2,000	428	311	3,519	2,872	253	84,354
14	4,353	467	204	71	787	638	102	52,014

## 3. The data

The IMSS (Mexican Institute for Social Insurance) had about 8 million insured in February 2022. The IMSS used SINOLAVE (Online Surveillance System for Influenza Epidemics) originally intended for influenza surveillance, as a COVID-19 surveillance system, which recorded 5,365,955 cases from 29/Dec/2019 to 01/Apr/2022. From this database we extracted fully anonymized data from vaccinated individuals with a posterior PCR positive test for SARS-CoV-2. Since the vaccination stage in Mexico started officially on 13/Jan/2021, all individuals (mostly healthcare workers) vaccinated before 13/Jan/2021 were recorded to be vaccinated on that date, thus, we included only individuals vaccinated after that date, which eliminated 97.5% of healthcare workers. For vaccinated individuals, we considered only the first confirmed infection in case an individual had two or more confirmed reinfections.

**Figure 1:**
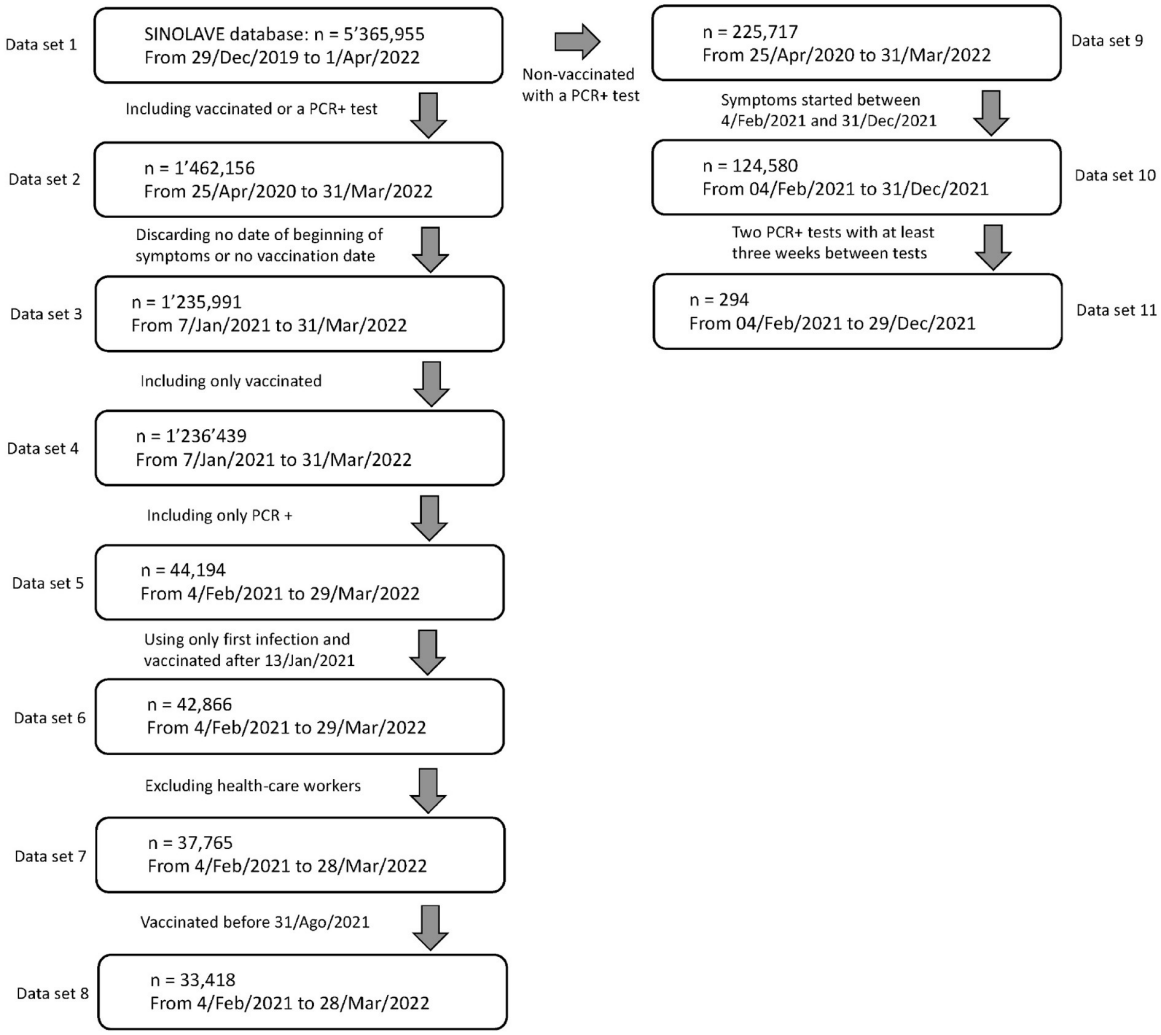
Data selection process from the original SINOLAVE database with 5,365,955 observations and 169 variables to our working data sets 8 and 11. Data set 8 is used to estimate the waning effect of vaccines whereas data set 11 that of active infection.

To reduce the effect of the Omicron variant, we only analyzed patients whose symptoms started between 4/Feb/2021 and 28/Mar/2022. A recently published study showed that the dominance of Omicron sublineages, waning of effectiveness against hospitalization is evident as early as 3–4 months after vaccination (22), even in fully immunized subjects. The resulting dataset (data set 8 in Figure 1) was used to characterize waning vaccine effect and it has 33,418 individuals.

To characterize the waning immunity of active infection, we considered all non-vaccinated individuals with two PCR+ confirmed infections with at least 21 days between tests, whose symptoms started also between 4/Feb/2021 and 31/Dec/2021, to avoid most of infections caused by the Omicron variant. The resulting dataset, Data set 11, was used to characterize waning vaccine effect and it has 294 PCR+ infections corresponding to 147 individuals. Figure 1 shows the data selection process, from the original data set to the working data sets 8 and 11.

Some basics statistics of the data sets 8 and 11 are shown as Supplementary Tables 1, 2. Table 3 shows statistics on the number of vaccinated individuals for each vaccine in our study.

**Table 3:** Number of infections observed for every vaccine.

**Vaccine**	**Doses**	Mean ± s.d.^*^	***N***	*t* ≥ 14^**^	**One dose**	**Two dose**
AZ	2	64.8 ± 32.7	13,772	12,194	4,688^†^	7,506
CA	1	0 ± 0	2,213	2,106	1,997	109^‡^
MO	2	63 ± 55.9	207	176	41^†^	135
SP	2	43.9 ± 28.6	195	183	43^†^	140
SV	2	42.8 ± 24.4	4,762	4,369	960^†^	3,409
GA	2	57.2 ± 33.8	1,475	1,333	328^†^	1,005
JA	1	0 ± 0	1,636	1,583	1,566	17^‡^
PF	2	42 ± 25.6	9,124	8,558	1,220^†^	7,338

A Weibull distributed was not fitted in those cases with < 100 observations.

* Mean and s.d. of the number of days between doses.

** Number of observations with at least 14 days from vaccination to infection.

† Individuals with incomplete dose.

‡ Individuals with more than the recommended dose.

## 4. Methodology

### 4.1. Justifying the use of non-censored observations exclusively

To estimate the waning vaccine effect, we use the times elapsed from vaccination to infection to fit a survival function for each vaccine. We use a surrogate for the time of infection as the date in which symptoms began as declared by PCR+ confirmed patients. Thus, our study naturally excludes two types of vaccinated individuals: (a) those individuals whose infection was not detected and (b) those individuals that were not infected between the time from first vaccination until 28/Mar/2022.

If the case is (a), estimating the parameters of a survival function is not affected for the following reason: fitting a survival function requires only elapsed times between times of vaccination and infection. A known result (23) states that if a random sample of the elapsed times is taken, and the sample is independent of the duration of the elapsed times, then, the estimation process is still unbiased. In our particular case, we can assume that most lost cases are due to mild symptoms, therefore, as long as the elapsed time between vaccination and infection is independent of the symptom type (mild, severe), we can apply the usual methodology to fit a survival function. The observed difference in the average time to infection from vaccination (all vaccines) among those with mild or severe infection groups is 5 days, and the hypothesis that the mean elapsed time from vaccination to infection between both groups is < 4 days is not rejected. Supplementary Figure 1 shows how the cumulative proportions of hospitalized (severe) and ambulatory (mild) cases evolved with time from vaccination. Although it is impossible to know the fraction of mild cases lost and when they were lost, it seems that at least for the first 10 months from vaccination the ratio mild to severe cases detected is preserved, which favors the argument that appearance of mild/sever cases is random and thus, mild cases are lost at random. Other factors like age, gender or known comorbidities are reflected in the severity of symptoms, thus, none of these factors should add a bias to the remaining sample.

If the case is (b), in which vaccinated individuals were not infected from the day of first shot to 28/Mar/2022, they may reflect a protective effect of a vaccine, and thus, avoiding them adds a bias to the estimate, since an individual will tend to be eliminated from the sample if a vaccine confers a great amount of protection for a long time. There is a way to reduce the bias caused by this kind of censorship: chose a value of t^*^ large enough so that there is a high certainty that no vaccine protection exceeds t^*^ and avoid the observations that were not followed for at least t^*^ days, unless the event occurred before t^*^. The amount of bias introduced by this method depends on how large is t^*^, and the bias is 0 if *t* → ∞ or if we wait until all vaccinated are infected. Since the largest time elapsed between the application of the first vaccine in our working database and the appearance of symptoms in our data is 426 days, this is the maximum value we can choose for t^*^, which is a reasonable amount of time for the vanishing of most vaccine effect, according to the literature. Thus, in essence, we are including only observations with outcome in the following 426 days after first vaccination, which requires to eliminate all vaccinated with no detected infection, which either were not infected before t^*^ or did not fulfill the required t^*^ units of observation.

### 4.2. The effect of natural immunity on the survival analysis

The method used here is robust to the presence of individuals with natural immunity among the vaccinated. That is, if there are individuals which are immune to the disease among those vaccinated, the waning effect of vaccines can still be parameterized. We assume that the vaccine provides some amount of initial protection against infection and with time this protection wanes and infections will start occurring among those vaccinated. If there are immune individuals among vaccinated, they are still immune when the vaccine effect wanes completely.

### 4.3. Measuring vaccine performance

We opted for fitting the same survival function to all vaccines, with comparison purposes. We chose the survival function that best fitted all of them, suggesting a failure time according to an AFT Weibull distribution, with *cumulative hazard function* λ(*t*) = (λ*t*)^*k*^. The AFT Weibull distribution is a versatile distribution that has been extensively used in destructive processes in industry and medicine (24). In here, we will obtain maximum likelihood parameter estimates (MLE's) of λ and *k*. Observe that the times of infection of two individuals, *t*_*i*_ and *t*_*j*_, may be correlated, but they are independent, which is essential for estimation purposes.

It is important to emphasize that this analysis only allows to characterize how the protective effect wanes with time, but not the amount of initial protection conferred by the vaccine. A vaccine may induce a stronger protection than another at the beginning, but its protective effect may wane faster, and, at the end, it could offer less total protective effect. Observe that:


$$\begin{array}{l} P_{v}\left(T>t\right)=S_{v}\left(t\right) \end{array}$$

is the probability that an individual receiving vaccine *v* is not infected in the next *t* days after first vaccination. With this, we suggest using as a measure of performance the index:


$$\begin{array}{l} \int_{0}^{\infty }S_{v}\left(t\right)\;dt \end{array}$$

which is the total protection conferred by vaccine *v* during its lifespan. Observe that by definition, the previous integral is the expected value of *T*. Therefore, the average time to infection under vaccine *v* will be used as a measure of performance.

### 4.4. Characterizing waning immunity due to active infection

The main database contains information on elapsed time between infections for non-vaccinated individuals, which can be used to characterize any immunity effect in a similar fashion to those vaccinated. To reduce the effect of infections due to Omicron variant, we use data from individuals whose symptoms started prior to 31/Dec/2021. Data set 11 (see Figure 1) contains non-vaccinated individuals with two infections whose symptoms of the first infection began between 4/Feb/2021 and 31/Dec/2021. We required a PCR+ test for both infections and at least 21 days between them. The resulting data set has 294 infections from 147 individuals.

### 4.5. Characterizing immunity against severe disease

The immune efficacy due to infection or vaccination has three components: the reduction in susceptibility to infection, the reduction in infectiousness and the reduction in pathology (25). In this work we attempt to estimate how vaccination affects the severity of the disease, by fitting a logistic model with age as independent variable and severity of symptoms as dependent. Our database only includes information on who was hospitalized and who was sent home (ambulatory), and we will use this is a surrogate of severity of symptoms. Since we also have the age of those unvaccinated at the time of first infection, we will also fit a logistic model to this group with comparison purposes.

## 5. Study limitations

In order to build a survival model for the time to response under a particular treatment (vaccine), it is necessary that all individuals that receive a particular vaccine, are subjected to the same hazard function λ(*t*), but the differential exposure does not provide these conditions: along the evolution of the epidemic, there are times in which the infection rate increases, which manifests in “waves” in the number of infections. The likelihood that a vaccinated person is infected on a given day, depends on two factors: (a) the amount of pressure of infection on the individual, which is some function of the number of infectious around, and (b) the remaining protective effect of the vaccine. When estimating the waning effect of a vaccine the variability in the infection pressure may affect our estimate of how the vaccine wanes. For instance, 84% of healthcare workers in the database received PF vaccine and 11% AZ. Since there is evidence that shows that healthcare workers have a risk of infection about three times higher than non-healthcare workers (26) this may jeopardize how the vaccine effect wanes for some vaccines. To reduce the amount of differential exposure, we excluded healthcare workers from the database. As mentioned before, most of the health workers (97.5%) had already been eliminated because the vaccination date was unknown.

The differential deployment of vaccines is also important. Define *vaccine age* as the current time from vaccination with the first dose of a particular individual. In Supplementary Figure 2, we show how the average vaccine age relates to a surrogate of the infectious pressure, as it is the accumulated percentage of infections during the study time. The *x*-axis is the accumulated percentage of infections and the *y*-axis is the average vaccine age. Had all vaccines been subjected to the same infectious pressure, the lines would overlap. There is no way to control this differential exposure, therefore, before the modeling stage, it is impossible to know if this is a determinant factor, nevertheless, as it will be shown later, this effect seems to be relatively small.

## 6. Results

### 6.1. Active infection

**Figure 2:**
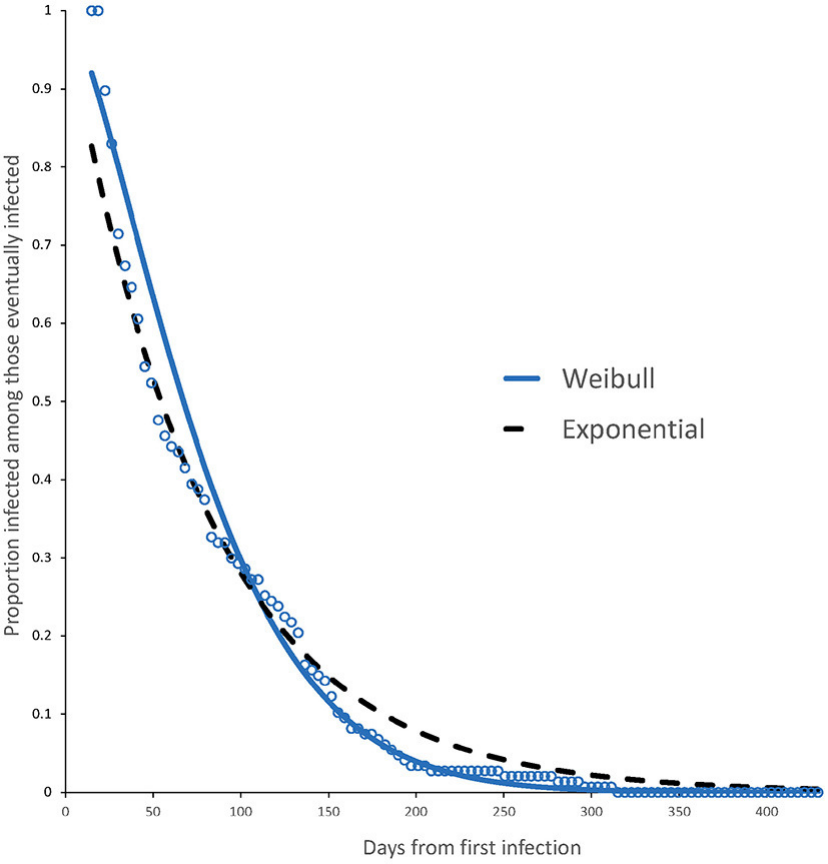
Survival curve and fitted model (Weibull, continuous line) for individuals with immunity from active infection. The plot also shows the fitted exponential model (dashed line) for comparison purposes. The Weibull model has a better fit for later stages of immunity whereas the exponential model fits well at earlier stages. Both models have the same average protection time.

Figure 2 shows how immunity due to natural infection decays with time. We also fitted an exponential decay model with comparison purposes. Both models lack of fit at one end of the data: whereas the Weibull model fits well later stages of the immunity decay, the exponential model better fits the decay at earlier stages. We will use these models to compare the reported decay in antibodies due to active infection in Section 7. Both models have the same average protection (Weibull: 79.4 d, Exponential: 78.7 d).

### 6.2. Complete vs. incomplete dose

In this section we analyze observed vs. fitted survival curves for each vaccine, considering whether the individuals completed the recommended number of doses or not. Table 3 shows the amount of data available for each group (last two columns). The table reveals that we can analyze the effect of a complete dose in all eight vaccines but only four allow to study the effect of an incomplete dose, since we did not fit a model in groups with < 100 observations. For vaccines CA and JA, with a single recommended dose, we excluded individuals with more than one dose.

Table 4 shows the parameters for the fitted Weibull model for complete and incomplete dose for each vaccine and for the active infection. We can see that, on average, the mean protection time of those receiving complete dose is 2.6 times larger than those receiving incomplete dose, and 2.2 times that of active infection. Although not shown in Table 4, we can see that the mean protection time of active infection is 1.1 times larger than the protection conferred to those with incomplete dose, that is, they are similar. Confidence intervals for the parameters are provided as Supplementary Table 3, and a summary of how the protection wanes is shown in Table 5.

**Table 4:** Fitted parameters for the survival function *S*(*t*) for a Weibull distribution (λ, *k*) for complete and incomplete dose, with at least 100 observations.

**Vaccine**	**Complete dose**				**Incomplete dose**			
	***N*_1_**	**λ_1_**	***k*_1_**	$\mu_{1}^{*}$	***N*_0_**	**λ_0_**	***k*_0_**	$\mu_{0}^{*}$	**μ_1_/μ_0_^†^**	** $\mu_{1}/\mu_{AI}^{‡}$ **
AZ	7,506	205.6	2.9	183.4	4,688	65.6	1.3	60.2	3	2.3
CA	1,997	166	2	147.1	–	–	–	–	–	1.9
MO	135	217	3.6	195.6	41	–	–	–	–	2.5
SP	140	191	2.7	169.9	43	–	–	–	–	2.1
SV	3,409	184.9	2.5	164.1	960	70.1	1.2	66.7	2.5	2.1
GA	1,005	206.2	2.9	183.8	328	77.5	1.2	72.4	2.5	2.3
JA	1,566	178.6	3	159.4	–	–	–	–	–	2
PF	7,338	235.3	2.7	209.2	1,220	92	1.1	88.6	2.4	2.6
AI	147	87.3	1.4	79.4	–	–	–	–	–	1

The data used was elapsed time from first dose. Last row contains parameters for the duration of immunity due to active infection (AI).

AI, active infection.

${}^{*}\mu_{i}=\lambda \;\Gamma \left(1+k\right)$, from fitted Weibull model.

^†^μ_1_/μ_0_, the ratio complete vs. incomplete dose average protection time.

${}^{‡}\mu_{1}/\mu_{0}$, the ratio complete vs. active infection average protection time.

**Table 5:** Day at loss of a given protection ability of vaccines among those receiving complete dose with at least 100 observations.

**Vaccine**	***N***	**Proportion of protection lost**				
		**0.5**	**0.6**	**0.7**	**0.8**	**0.9**
AZ	7,506	181	199	219	242	274
CA	1,997	138	159	182	211	252
MO	135	196	212	228	248	274
SP	140	167	185	205	228	260
SV	3,409	160	179	199	224	258
GA	1,005	182	200	220	243	275
JA	1,566	158	173	190	209	236
PF	7,338	205	228	252	281	320
AI	147	67	82	100	122	157

Calculated from the fitted Weibull distribution. Days from first dose.

AI, active infection.

**Figure 3:**
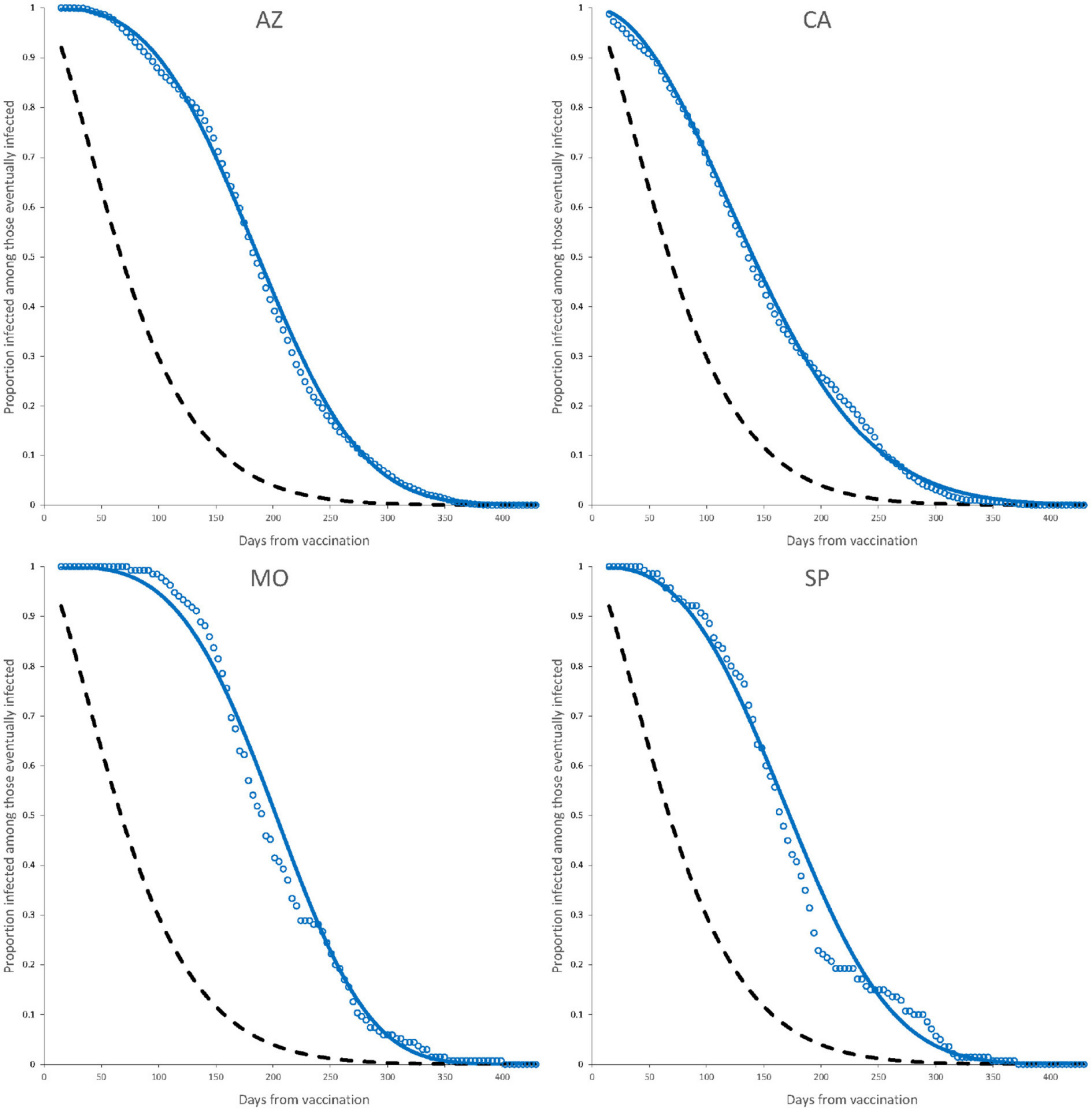
Observed elapsed time to infection (dots) and fitted model (continuous line) for complete dose vaccines AZ, CA, MO, and SP. Dashed line is the fitted Weibull model for active infection shown in Figure 2. Parameter estimates are those of a complete dose shown in Table 4.

**Figure 4:**
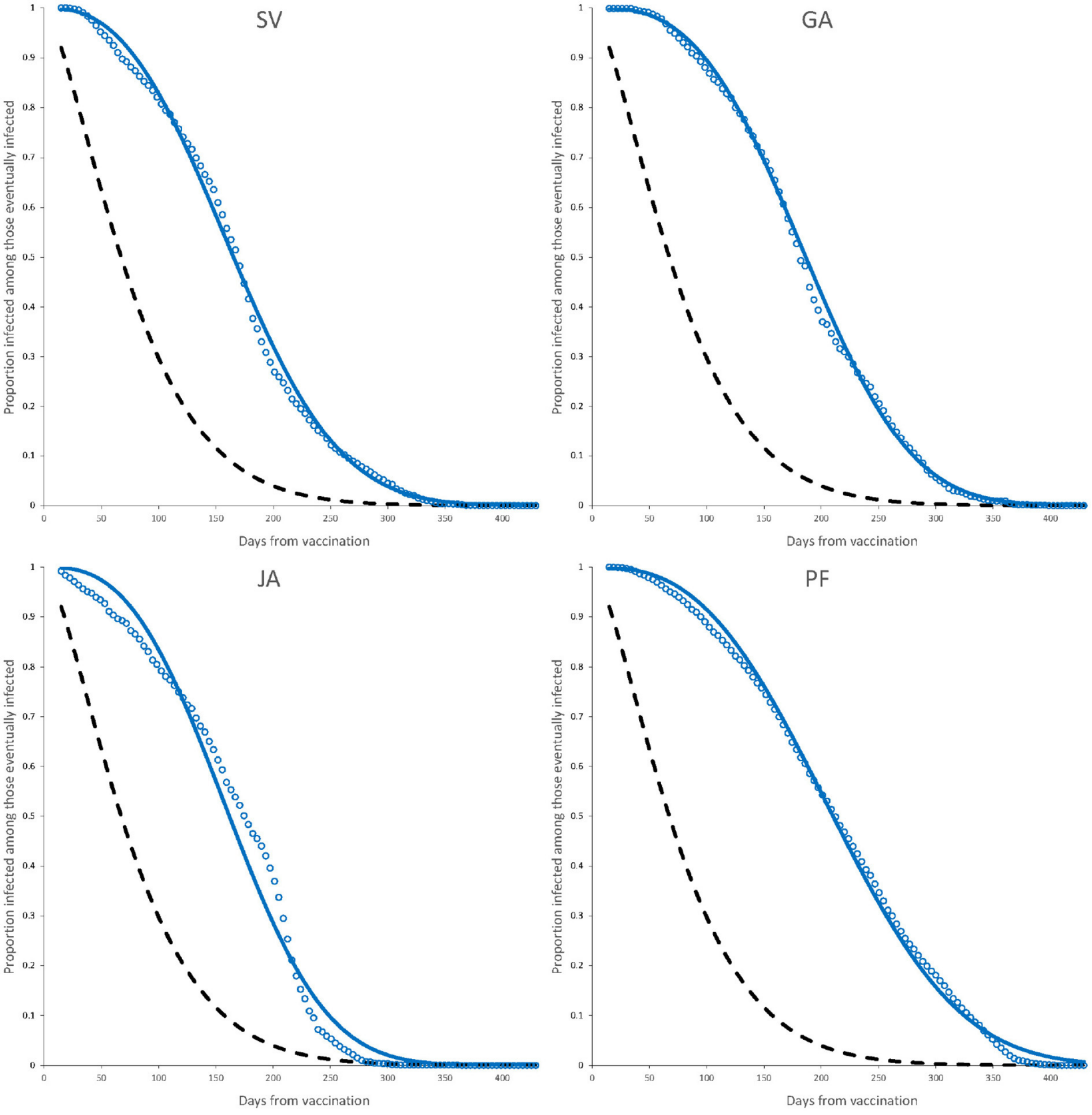
Observed elapsed time to infection (dots) and fitted model (continuous line) for complete dose vaccines SV, GA, JA, and PF. Dashed line is the fitted Weibull model for active infection shown in Figure 2. Parameter estimates are those of a complete dose shown in Table 4.

The observed data together with the fitted Weibull survival distribution as well as the adjusted Weibull model for active infection, are shown in Figures 3, 4.

### 6.3. Age < 60 vs. age ≥ 60

**Figure 5:**
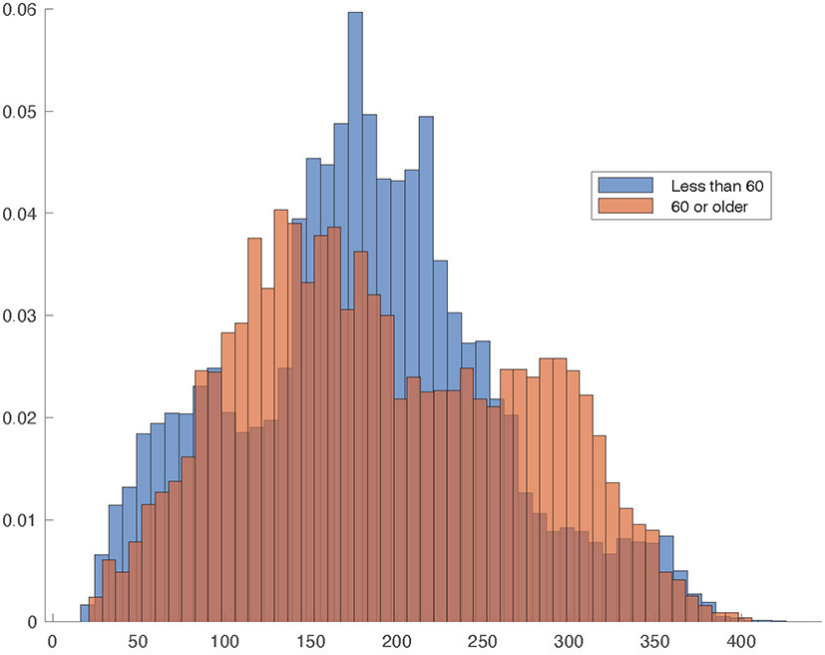
Relative frequency histogram of the elapsed times between vaccination and day of beginning of symptoms for age < 60 and age ≥ 60. The darker area is the overlapping region.

No difference is observed in the distribution of the elapsed times for both age groups, all vaccines combined. The median of the elapsed times between vaccination and the day of beginning of symptoms is 180 days for those with age < 60 and 181 days for those with age ≥ 60. Figure 5 shows the relative frequency histogram of the elapsed times for both groups. Within vaccines, the difference in medians is smaller 12 days for each vaccine.

### 6.4. Vaccine protection against severe disease

**Figure 6:**
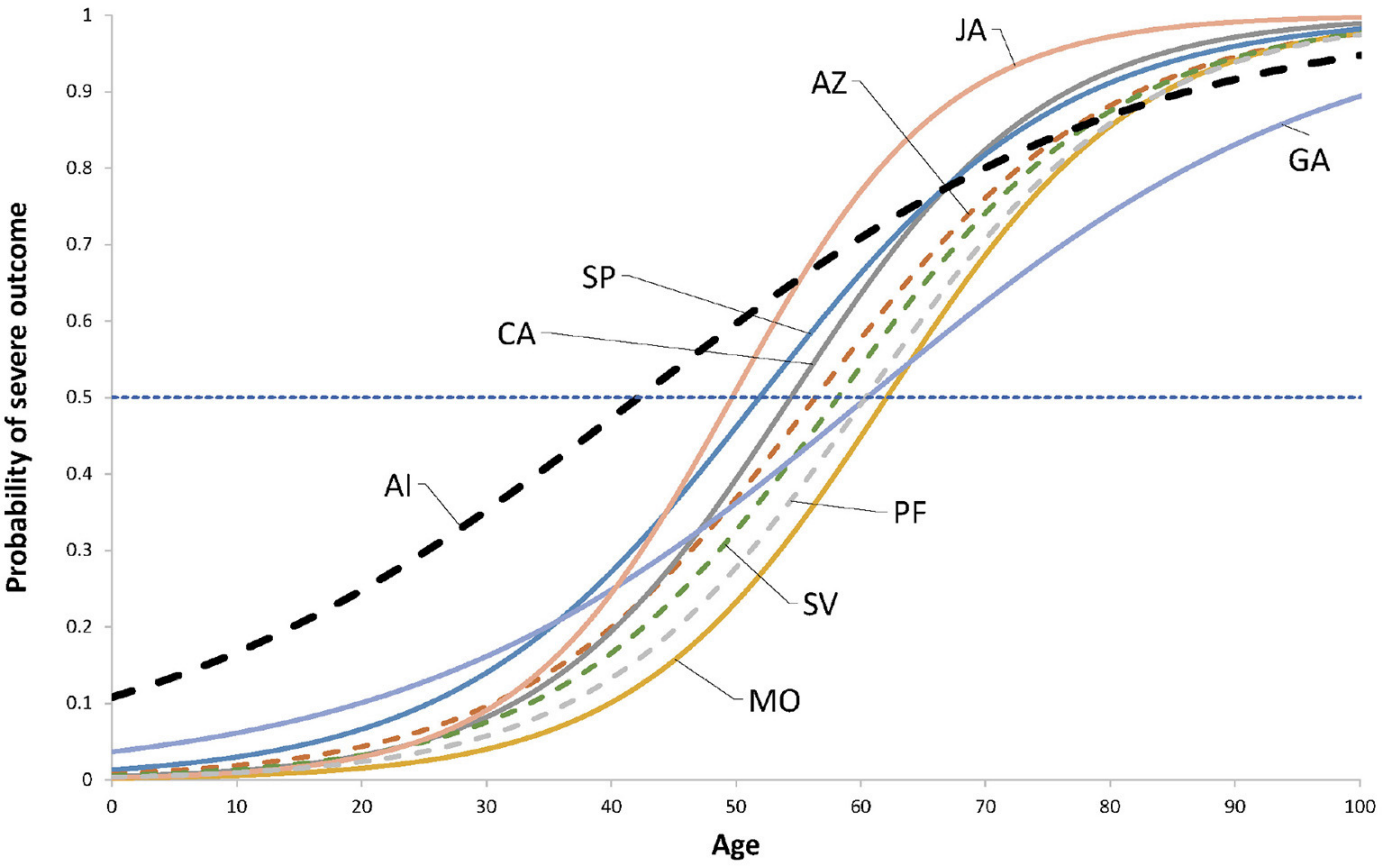
Fitted logistic models 1/[1 + exp(− β_0_ + β_1_ * Age)]. The coefficients are shown in Table 6. The horizontal line show the 50% probability. Dashed lines indicate vaccines where the model has a poor fit to data according the Hosmer-Lemeshow test. The dashed thick line to the left is the fitted model for the outcome of the first infection of those unvaccinated.

A well-known comorbidity for infection with SARS-CoV-2 is age. We analyzed the effect of age on the likelihood of a severe outcome (resulting in hospitalization) for all vaccines. We compare this behavior with the outcome of the first infection in unvaccinated individuals, using a logistic model. Table 6 contains the estimated parameters and Figure 6 shows a plot of the fitted logistic models.

**Table 6:** Parameters of the logistic model 1/[1 + exp(−(β_0_ + β_1_ * Age))] for each vaccine.

**Vaccine**	**β_0_**	**β_1_**	${\text{Age}}_{50}^{*}$	**Dif^†^**	***p* ^‡^**
AZ	−4.791	0.085	56.4	14.2	0.001
CA	−5.385	0.099	54.4	12.2	0.205
MO	−6.146	0.099	62.1	19.9	0.588
SP	−4.306	0.083	51.9	9.7	0.933
SV	−5.178	0.089	58.2	16.0	0.013
GA	−3.268	0.054	60.5	18.3	0.824
JA	−5.814	0.117	49.7	7.5	0.258
PF	−5.557	0.029	60.4	18.2	0.001
AI	−2.110	0.050	42.2	0.0	0.001

AI corresponds to the first infection for unvaccinated individuals. Parameters β_0_ and β_1_ were significative (*p* < 0.001) for all vaccines and AI.

^*^Age at which there is a 50% probability of severe disease if infected. This is − β_0_/β_1_. ^†^Difference with ${\text{Age}}_{50}^{*}$ for AI.

^‡^Hosmer-Lemeshow test of goodness of fit. Models with a low *p*-value have a poor fit.

## 7. Discussion

Different individuals may react differently when the vaccine strength has been reduced, say, to 50%, depending on facts as comorbidities, amount of exposure, age, gender, etc. For some, a 50% reduction level represents still a high level of protection whereas for others it is already a huge loss, therefore, it is expected that the survival function depends on the characteristics of the population. Age structure and comorbidities play an important role. The methods used here can be applied to more specific categories as the ones described (age, complete/incomplete dose).

The main difference with other studies to characterize the waning effect of vaccines is that in this study we consider the time to failure, instead of a sample of attack ratios taken at regular intervals. This allows for a characterization of the survival curves *S*_*v*_(*t*). Visually, the Weibull model seems to fit well, especially considering those factors mentioned previously, as the potential effect of differential exposure, nevertheless, the Anderson-Darling test rejects the hypothesis that the underlying distribution is Weibull AFT (*P* < 0.001) for all vaccines.

The usefulness of the incomplete dose data depends on its nature: if the individual misses the second dose for a reason that is associated with exposure, as it is the case of individuals that may have lost interest in receiving the second dose or believe that they have enough protection already, those may be factors that increase the exposure (27) and thus the data on incomplete dose would be useless. If the lack of a second dose is independent from exposure or risk, then the incomplete dose model provides information on the increase achieved by observing the recommendations of the manufacturers. The ratio μ_1_/μ_0_ in Table 4 suggests that the increase in average protection time of a complete dose ranges between 2 and 3 times that of an incomplete dose, with an unweighted average between vaccines of 2.6.

Although we are measuring decay in vaccine protection, this decay should have a correspondence with VE of the form VE ∝*S*(*t*), so we performed some comparison with some VE estimates: Nordstrom et al. (28) obtained estimates of VE for several cohorts for vaccines PF, MO, AZ, and a mixture of AZ and MO. Unfortunately, the intervals are too width to be comparable in some cases. For instance, Nordstrom et al. (28) reports that AZ vaccine has a VE of 49% in the interval 31–60 day after second doses, and 41% in the interval 61–120 days. This implies that the 45% VE level is reached somewhere between 31 and 120 days. For PF vaccine, Nordstrom et al. (28) reports that an estimated VE of 47% was observed in the interval of 121–180 days after second dose. Using the complete dose model parameters in Table 4, PF vaccine loses 50% protective effect by day 163, which is in the range of 121–180 days reported by Nordstrom et al. (28).

Also, for PF vaccine, Nordstrom et al. (28) reports a VE of 23% after 210 days from second dose, whereas our Weibull model for the complete dose (Table 4) shows that by day 257 from first dose the remaining protection of the vaccine is 30%.

A larger contrast between Nordstrom et al. (28) and this study is related to the AZ vaccine, since they found this vaccine has lost the VE after 120 days from second dose, while our complete dose model suggests that, after subtracting the average time between first and second dose of 65 days, by day 122 of second dose AZ still exhibits a 48% protective strength.

Our results agree with those of Andrews et al. (19), where it is reported that by day 140 after second dose, the vaccines AZ and PF vaccines have reduced the VE to 44.3 and 66.3%, respectively. When using the survival curve from our fitted Weibull model for the complete dose, the protective efficacy of the vaccines by day 140 has been reduced to 37 and 60% for AZ and PF vaccines, respectively.

Our results also agree with those of Bedston et al. (20), who calculated a VE of 86% by day 14 and of 53% by day 154 for PF vaccine after second dose. Our survival model for complete dose PF vaccine for those days is 97 and 54%, respectively.

In a study by Menni et al. (29), with 620,793 participants of the ZOE COVID Study (30) the VE of vaccines AZ, MO and PF was measured at 5 months from second dose, and the respective VE's were 75.7, 84.3, and 82.1%. These results are optimistic compared to Nordstrom et al. (28), Andrews et al. (19), Bedston et al. (20) and our own results here, since using the complete dose model with the parameters in Table 4, after fitting the average time from first and second dose, we have respectively 32, 38, and 56% remaining vaccine effect, 5 months after second dose. It is difficult to assess the effect of voluntary participation when uploading information to the platform described in Menni et al. (31).

Khoury et al. (7) and Padmanabhan et al. (11) analyzed the mean neutralization level of antibodies against SARS-CoV-2 (NT_50_, the dilution at which the neutralization efficiency of the plasma decreases by 50%) and related this to estimates of vaccine efficacy. Table 7 shows the Spearman's correlation between our estimate of mean protection time from Table 4 and NT_50_ and the two measures of VE used in Khoury et al. (7) and Padmanabhan et al. (11). By analyzing the vaccines that are common in those studies and this work (SV, JA, AZ, GA, MO, and PF) we can see that μ_1_ is highly correlated with those measures, and the decrease in correlation of μ_1_ from VE_*P*_ to VE_*K*_ is due only to a difference in both studies in the VE attributed to AZ vaccine, 80 vs. 61%.

**Table 7:** Spearman's correlation between NT_50_, average protection time (μ_1_) and the measures of VE used in (11) (VE_*P*_) and (7) (VE_*K*_).

	**μ_1_**	**NT_50_**	**VE_*P*_**	**VE_*K*_**
μ_1_	1.00	0.94	0.89	0.83
NT_50_		1.00	0.94	0.94
VE_*P*_			1.00	0.89
VE_*K*_				1.00

Varona et al. (12) built a model for SARS-CoV-2 IgG antibodies decline and we compare this with the decay in immunity from active infection shown in Figure 2. The model of decay is *f*(*t*) = 6.4643exp(− α *t*) with α = 0.003754. Since the average time to infection after the first infection is 79.4 days (see Table 4) then the average a.u. concentration at infection is [*f*(79.4) − 1.1]/(6.4643 − 1.1) = 0.689, that is, at 69% of the a.u. concentration at baseline. This clearly is not a constant and will depend on the infection pressure.

Regarding the protection against severe disease, it seems that vaccination increases the age at which those infected reach a 50% probability of developing severe symptoms. The increase ranges from 7.5 (JA) to 20 (MO) years, with an unweighted average increase of 15.5 years (median 15.1). Most vaccines have similar slope (β_1_) except GA, whose slope resembles that of the active infection. The mean Age_50_ is 56.7 years (median 57.3).

Observe that the likelihood of severe disease seems to be lower for unvaccinated individuals in those with age 80 or older (Figure 6), but this must be taken carefully since three vaccines (AZ, SV, and PF), besides the AI model, have poor fit. This may be due to scarce data in this age group, especially for the AI. These models may provide information on approximate Age_50_ values, but they may be considerer only as approximations.

**Figure 7:**
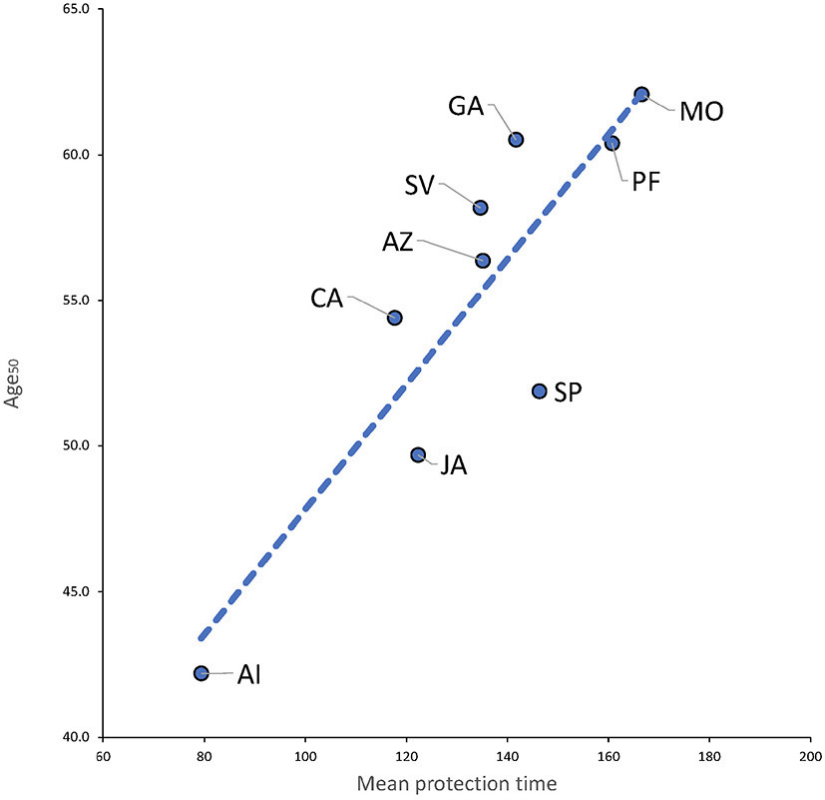
Plot of mean protection time (μ_1_) vs. Age_50_. *R*^2^ = 0.765.

Figure 7 shows that μ_1_, the mean protection time is associated with larger values of Age_50_. This does not imply that a longer elapsed time between vaccination and infection will most likely result in mild symptoms, but only that a vaccine with larger μ_1_ protects individuals against severe disease more efficiently, and that the protection is positively correlated with age. For instance, consider vaccine MO (selected because it is the vaccine with the largest Age_50_ with a good fit). The average time to a mild (severe) outcome is 196.12 (195.32) days (N.S. *p* > 0.74) that is, they are similar, nevertheless, this vaccine increases Age_50_ in 20 years on average.

A graphical analogy will help explain these results: imagine a series of points in a timeline, corresponding to the elapsed times from vaccination to infection. Points may be white or black corresponding to mild and severe cases of disease, respectively. Let the average of the elapsed times be $\bar{X}$. What vaccines are doing is taking a sample of black dots and converting them to white (corresponding more likely to older individuals), which does not change $\bar{X}$, only Age_50_.

It is important to notice that in our study there were 1, 528 vaccinated individuals with a *vaccine age* larger than 400 days and of these, only 40 (2.6%) gave a positive PCR in that interval and out of these later group, 4 (10%) where hospitalized. There is an interesting interpretation of these facts, since they show that when the vaccine effect had reasonably waned for all vaccines, only < 0.3% required hospitalization. Given the high infectiousness of the different SARS-CoV-2 variants, this may suggest that there may be a large proportion of individuals with natural immunity in the population.

## 8. Conclusions

All evaluated vaccines had lost most of their protective effect between 8 and 11 months of application of first shot. The waning effect resembles an AFT Weibull distribution with a median life of 183 days from first shot. For vaccines with two doses, the mean protection time increases 2–3 times for individuals receiving a complete dose compared to those with an incomplete dose. The average vaccine protection is about 2.2 times larger than that of the protection conferred by active infection. The model for the decay of protection of active infection could fit an exponential decay, similar to the reported decay in antibodies but at different rate. The mean time to protection μ_1_ obtained in this study correlates very well with NT_50_ and vaccine efficacy reported elsewhere. The logistic model shows that vaccination increases Age_50_, the age at which there is a 50% probability of severe disease if infected, in 15 years on average, when compared with Age_50_ of active infection among those unvaccinated. Mean protection time μ_1_ is associated with larger values of Age_50_.

## Data availability statement

The data analyzed in this study is subject to the following licenses/restrictions: data are available from the authors upon reasonable request. Requests to access these datasets should be directed to EM-Z, efren.murilloza@imss.gob.mx.

## Ethics statement

The studies involving human participants were reviewed and approved by Local Health Research Ethics Committee 601. The patients/participants provided their written informed consent to participate in this study.

## Author contributions

CH-S: conceptualization, methodology, data curation, and writing—original draft preparation. EM-Z: conceptualization, data curation, and writing—original draft preparation. Both authors contributed to the article and approved the submitted version.

## Conflict of interest

The authors declare that the research was conducted in the absence of any commercial or financial relationships that could be construed as a potential conflict of interest.

## Publisher's note

All claims expressed in this article are solely those of the authors and do not necessarily represent those of their affiliated organizations, or those of the publisher, the editors and the reviewers. Any product that may be evaluated in this article, or claim that may be made by its manufacturer, is not guaranteed or endorsed by the publisher.
