# Supplementary material for: Waning immunity to SARS-CoV-2 following vaccination or infection
Source: Front Med (Lausanne). 2022 Oct 13;9:972083. doi: 10.3389/fmed.2022.972083 (PMC9606629; doi:10.3389/fmed.2022.972083)
Supplement: Supplementary file 1 [file Data_Sheet_1.PDF]

Supplementary Material

1 SUPPLEMENTARY TABLES

2 SUPPLEMENTARY FIGURES

Table S1. Age frequencies of vaccinated individuals used to characterize the waning effect of vaccines (data set 8 in Figure 1) by gender and outcome.

Age category		Gender		Hospitalized			Ambulatory
L	U	Male	Female	Dead	Recovered	n.d.*	
-	15	12	14	-	5	-	21
16	30	2,394	3,739	50	406	55	5,622
31	59	8,533	9,713	1,473	2,607	272	13,894
60	-	5,127	3,886	3,746	2,866	366	2,035

*Non-determined up to 29/Mar/2022.

Table S2. Age frequencies of unvaccinated individuals used to characterize the waning effect of vaccines (data set 11 in Figure 1) by gender and outcome.

Age category		Gender		Hospitalized			Ambulatory
L	U	Male	Female	Dead	Recovered	n.d.*	
-	15	8	14	0	16	1	5
16	30	46	31	5	14	1	57
31	59	57	63	9	36	6	69
60	-	27	48	10	48	4	13

*Non-determined up to 29/Mar/2022.

Table S3. Estimates and 95% CI for the parameters of the Weibull distribution. Complete dose and at least 100 observations. Last row contains parameters for the duration of immunity due to active infection (AI).

Vaccine	$\hat{\lambda}$	$\hat{\lambda}_L$	$\hat{\lambda}_U$	$\hat{k}$	$\hat{k}_L$	$\hat{k}_U$
AZ	205.63	203.96	207.31	2.92	2.87	2.97
CA	166.03	162.27	169.87	2.02	1.95	2.09
MO	216.97	206.57	227.88	3.64	3.22	4.12
SP	191.00	179.18	203.59	2.74	2.42	3.11
SV	184.93	182.35	187.55	2.51	2.45	2.58
GA	206.18	201.54	210.93	2.86	2.73	3.00
JA	178.60	175.52	181.74	2.96	2.83	3.08
PF	235.27	233.18	237.39	2.68	2.63	2.73
AI	87.29	77.31	98.55	1.41	1.25	1.59

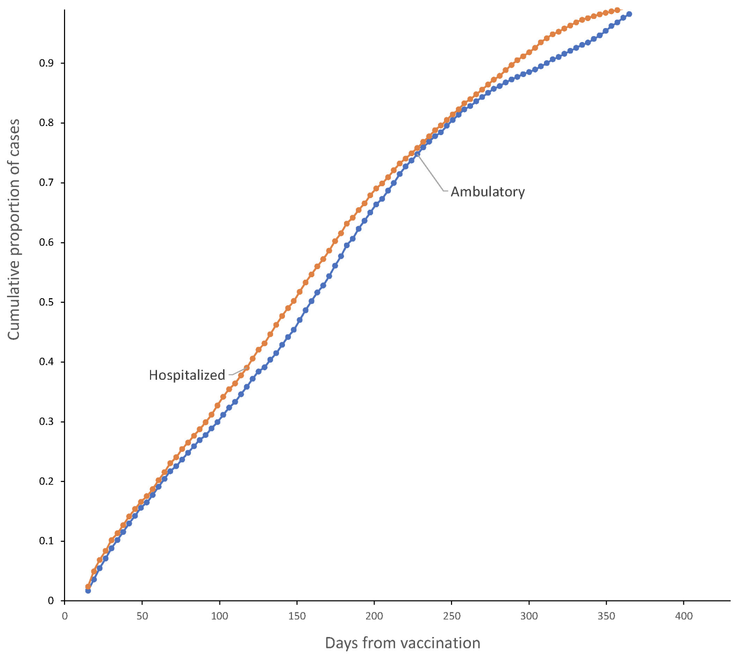

Figure S1. Evolution of the cumulative proportions of hospitalized (red) and ambulatory (blue) among the vaccinated infected, as a function of time from vaccination. The plot suggests that the severity of symptoms is independent of time from vaccination, thus, undetected cases (mostly mild cases) are lost at random and do not affect the characterization of how a vaccine wanes.

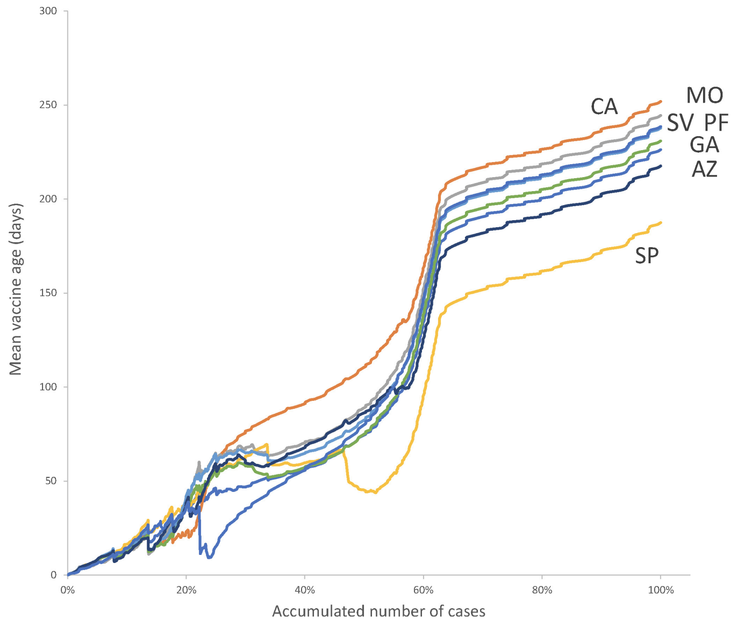

Figure S2. Mean age of vaccines vs. accumulated percentage number of cases in Mexico, this later used as a surrogate of exposure. The baseline in x-axis is 13/Jan/2021 (0%) and ends in 17/Feb/2022 (100%).
